# Pyogenic liver abscess after Milligan–Morgan hemorrhoidectomy

**DOI:** 10.1093/jscr/rjae388

**Published:** 2024-06-03

**Authors:** Jie Yang, Liman Zhang, Qiang Wang

**Affiliations:** Anorectal Department, Shijiazhuang Traditional Chinese Medical Hospital, Shijiazhuang 050001, China; Anorectal Department, Shijiazhuang Traditional Chinese Medical Hospital, Shijiazhuang 050001, China; Anorectal Department, Shijiazhuang Traditional Chinese Medical Hospital, Shijiazhuang 050001, China

**Keywords:** pyogenic liver abscess, Milligan-Morgan, hemorrhoidectomy

## Abstract

Milligan–Morgan(M-M) hemorrhoidectomy is regarded as the primary treatment option for patients diagnosed with grade III or IV hemorrhoids. Here, we present the case of a 50-year-old male who developed pyogenic liver abscesses as an unusual complication following M-M hemorrhoidectomy. Severe complications subsequent to hemorrhoid surgery are infrequent. A review of the PubMed database spanning the 30-year period between 1994 and 2024 yielded only four publications documenting patients who experienced liver abscesses following open hemorrhoidectomy. Furthermore, the patient exhibited symptoms of a liver abscess as early as the second day post-surgery, despite having no history of diabetes or liver disease, making this occurrence truly uncommon.

## Introduction

Pyogenic liver abscess (PLA) is characterized by high mortality and recurrence rates [1]. Six primary pathways for PLA infection have been identified [2]: the biliary tract, portal vein, direct invasion, hepatic artery, open wound, and cryptogenic pathways. Milligan–Morgan (M-M) hemorrhoidectomy is a globally recognized surgical technique for treating hemorrhoids.However, theoretically speaking, this surgery involves significant trauma and a prolonged healing time, which may lead to wound infections postoperatively, or bacteria entering the bloodstream could result in infections elsewhere, such as PLA. In this study, a 50-year-old male who underwent M-M hemorrhoidectomy suddenly presented with a high fever and abdominal pain 2 days postoperatively. Based on his symptoms, examination findings, and ultrasound results, PLA was suspected. This article discusses the diagnosis and treatment of a patient who developed secondary PLA following M-M hemorrhoidectomy, aiming to offer valuable insights for managing similar cases.

## Case report

**Figure 1:**
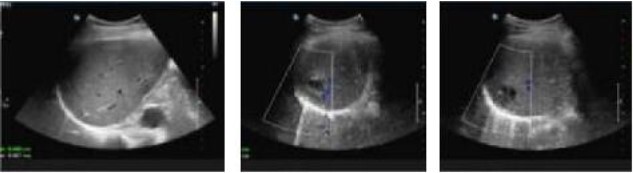
Abdominal color ultrasound findings on the third day after surgery.

A 50-year-old male patient is diagnosed with mixed hemorrhoid. He reported no prior medical history of hypertension; diabetes; cardiovascular, pulmonary, hematological disorders. The patient’s symptom was the prolapse of the anal mass after each stool and lasted for >2 years. Proctoscopy revealed the presence of two large external hemorrhoids with three internal hemorrhoids in the 3, 7, and 11 o’clock position. The patient underwent hemorrhoidectomy under combined lumbar epidural anesthesia. The surgery went smoothly. Unfortunately, on the second day post-surgery, the patient developed sudden chills and fever, with a body temperature of 39.9°C. Urgent investigations revealed the following: routine blood analysis showed white blood cells at 13.28 × 10^9/L, platelets at 307 × 10^9/L, lymphocyte percentage at 8.1%, and neutrophil percentage at 89.2%. The patient received intramuscular lyserpyrine injection (0.9 g), fluid rehydration, and physical cooling. Consequently, the patient’s body temperature decreased to 37°C. On the third morning post-surgery, the body temperature rose to 38.5°C. Further routine blood analysis revealed the following critical values: white blood cell count, 35.3 × 10^9/L, lymphocytes, 1.9%; and neutrophils, 95.2%. Examination of the wound tissue revealed no purulent exudate. The patient reported upper abdominal pain accompanied by nausea and reduced appetite. A prompt abdominal color ultrasound examination indicated a cystic solid echo near the right lobe of the liver, adjacent to the diaphragmatic apex, measuring ~29 × 26 mm, with indistinct borders. The diagnostic impression from the abdominal color Doppler ultrasound suggested a hepatic abscess (Fig. 1).

**Figure 2:**
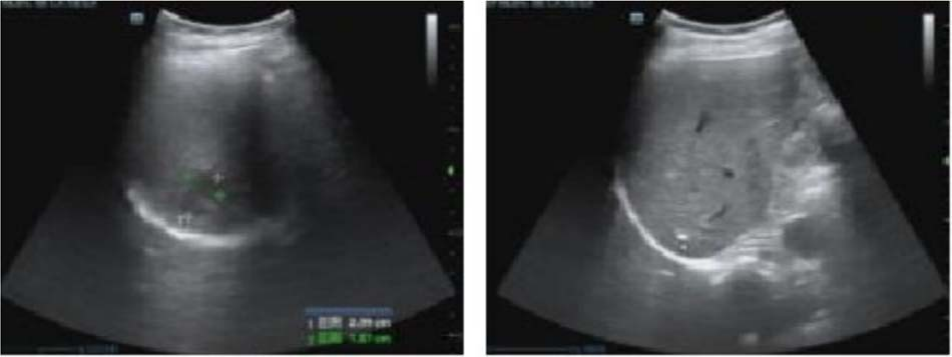
Abdominal color ultrasound findings on the 12th day after surgery.

The patient received anti-infective therapy comprising imipenem and cilastatin sodium along with a high-calorie digestible diet. By the sixth day post-surgery, the patient exhibited normothermia, improved appetite, and alleviation of abdominal discomfort. Subsequent routine blood analysis revealed a white blood cell count of 11.79 × 10^9/L, lymphocyte percentage of 21.3%, and neutrophil percentage of 70.7%. Re-evaluation using abdominal color ultrasound on postoperative Day 12 revealed that the extent of the abscess in the right hepatic lobe was reduced to 24 mm × 19 mm (Fig. 2).

Routine blood analysis indicated a white blood cell count of 8.74 × 10^9/L, lymphocyte percentage of 19.6%, and neutrophil percentage of 71.7%. Subsequently, imipenem and cilastatin sodium were discontinued, and oral therapy comprising cefixime dispersible tablets and metronidazole tablets was initiated for a duration of 1 week. The patient was discharged from the hospital with significant healing of the anal wound.

**Figure 3:**
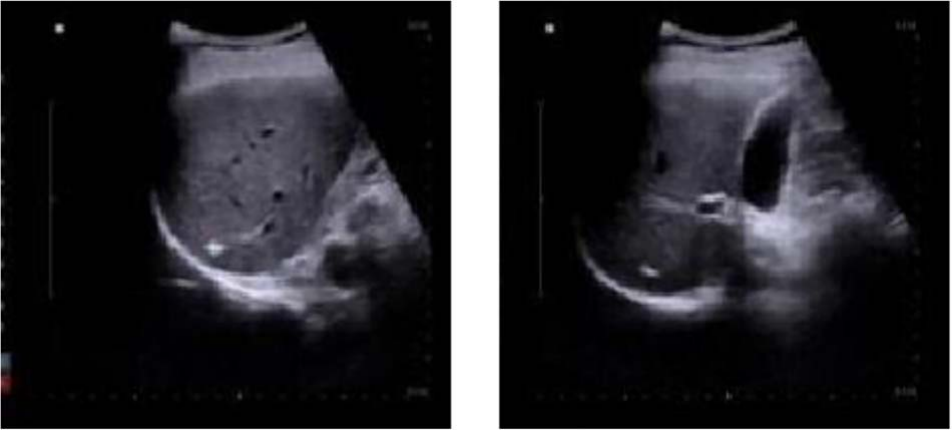
Abdominal color ultrasound findings on the 30th day after surgery.

One week after discharge, the patient underwent a follow-up examination at the outpatient department, which revealed significant healing of the surgical wound. The patient reported a normal appetite, absence of fever, and no abdominal pain or discomfort. Additionally, 1 month post-operation, a review of abdominal color ultrasound demonstrated the absence of inflammatory lesions in the liver (Fig. 3).

## Discussion

PLA accounts for 80% of liver abscess cases, with a case fatality rate of ~5.6% [3]. Early detection remains challenging, and severe cases can escalate to sepsis and septic shock, thereby posing grave threats. Fever is the predominant clinical indicator of PLA, often accompanied by chills, abdominal discomfort, nausea, and vomiting [4].

The current clinical literature on PLA post-hemorrhoidal surgery is limited [5], with some experts proposing a potential association with the migration of intestinal flora following necrosis and detachment of internal hemorrhoidal ligation tissue [6]. Furthermore, some scholars have linked PLA after hemorrhoidal ligation to factors such as dyslipidemia, digestive ulcers, and diabetes, hypothesizing transmural necrosis of the rectal mucosa and subsequent infection spread [7]. The timely diagnosis of PLA through abdominal color ultrasound follows the onset of epigastric pain and nausea, providing crucial guidance for clinical intervention [8]. In PLA management, empirical administration of broad-spectrum antibiotics is recommended for pathogen identification, with adjustments based on bacterial culture and drug sensitivity results [9]. Notably, Klebsiella and Escherichia are frequently reported as the primary PLA pathogens in China [10]. Considering the patient’s history of anal surgery, Escherichia intestinalis was deemed to be the most likely infective agent. Hence, a therapeutic regimen, including imipenem and cilastatin sodium, was employed to address potential *Klebsiella pneumoniae* infections, yielding satisfactory outcomes.

Maintaining strict adherence to sterility principles and ensuring thorough disinfection of the surgical site are paramount for reducing the risk of wound infection. Once PLA is diagnosed, it is crucial to administer antibiotics judiciously to combat the infection effectively. Additionally, for elderly patients with diabetes and compromised immune systems, extra precautions should be taken to prevent the development of such conditions, including the proper management of diabetes and measures to boost immune function. Early intervention and comprehensive care tailored to the needs of individual patients are key to ensuring favorable outcomes.

## Conflict of interest statement

None declared.
